# Long-term outcome in patients after treatment for Cushing’s disease in childhood

**DOI:** 10.1371/journal.pone.0226033

**Published:** 2019-12-12

**Authors:** Katarzyna Pasternak-Pietrzak, Elżbieta Moszczyńska, Marcin Roszkowski, Karolina Kot, Elżbieta Marczak, Wiesława Grajkowska, Maciej Pronicki, Mieczysław Szalecki

**Affiliations:** 1 Department of Endocrinology and Diabetology, The Children’s Memorial Health Institute (CMHI), Warsaw, Poland; 2 Department of Neurosurgery, The Children’s Memorial Health Institute (CMHI), Warsaw, Poland; 3 Pathology Department, The Children’s Memorial Health Institute, Warsaw, Poland; 4 Collegium Medicum, University of Jan Kochanowski, Kielce, Poland; Brown University Warren Alpert Medical School, UNITED STATES

## Abstract

**Introduction:**

Cushing’s disease (CD) is a rare cause of hypercortisolemia presenting a major diagnostic and therapeutic challenge. Data on pituitary function in long-term follow-up after CD treatment in childhood is limited.

**Aim:**

Long-term assessment of patients of the Children’s Memorial Health Institute (CMHI) after CD treatment in childhood.

**Materials and methods:**

Retrospective analysis of 29 CD patients, mean age at the time of diagnosis 13.46 yrs. The long-term follow-up (FU) was done by: 1) obtaining the data from a patient’s questionnaire (75% of adult patients); 2) using the data from the last clinic visit for patients who did not respond to the questionnaire and for current CMHI patients. The average long-term FU from transsphenoidal pituitary surgery (TSS) was 10.23 yrs.

**Results:**

At the latest FU: 18 patients (62%) had long-term disease remission after TSS1, 2 patients (6.9%) after TSS2, 1 patient (3.4%) after the post-TSS radiotherapy (XRT) cycle and 3 patients (10.3%) after bilateral adrenalectomy (BA). One patient (3.4%) died after TSS2 due to postoperative complications, 1 patient (3.4%) had persistent disease at latest FU, in 1 patient (3.4%) the long-term FU was not possible to perform. CD recurrence occurred in 4 out of 28 patients (14%) at an average time 3.6 yrs. from definitive treatment. One patient (3.4%) after BA was operated because of Nelson's syndrome. Two patients (6.9%) were suspected of relapse at latest assessment. At the time of the last evaluation, 17 patients (63%) were on levothyroxine therapy since definitive treatment, 16 patients (59%) were on hydrocortisone treatment, 10 patients (37%) were taking sex hormones replacement, 4 patients (15%)—antidiuretic hormone.

**Conclusions:**

Relatively large number of patients after CD treatment in childhood have hormonal pituitary deficits as well as mood and cognitive disorders. CD recurrence can occur even after a long time post effective treatment.

## Introduction

Cushing’s disease (CD) is characterized by excess production of adrenocorticotropic hormone (ACTH) by a pituitary corticotroph adenoma, which results in hypercortisolemia. As CD is extremely rare in the pediatric population, the disease is often challenging for physicians of different specialties.[1]. Thus, close cooperation in the diagnostic and therapeutic process between pediatric endocrinologists, pituitary neurosurgeons and pituitary radiotherapists is necessary [1]. Transsphenoidal pituitary surgery (TSS) is considered as the optimal treatment for CD and is a safe and effective method [2, 3]. The remission rates depend on the time from successful treatment: in the general population of CD patients (children and adults) remission rates are 45–98% in a short period from TSS and 50–98% in a long term follow-up (FU), however this depends on the criteria used to define cure of the disease [4, 5, 6, 7, 8, 9, 10, 11, 12, 13, 14]. Data for long-term assessment of pituitary function are limited in comparison to adult population [6, 7, 8, 5, 16, 17]. In this study we present the results of an analysis of 29 patients after CD treatment with the particular emphasis on the long-term FU.

## Patients and methods

Twenty-nine patients (15 girls and 14 boys) were diagnosed with CD and/or treated at Children’s Memorial Health Institute (CMHI), Warsaw, Poland between 1993 and 2018.

All patients underwent TSS as a primary treatment for CD (24 patients were operated by the same neurosurgeon at CMHI, 5 patients at other centers). Data was retrospectively collected and therefore some data is missing. The average age of all patients at surgery was 13.49 yrs. (5.42–17.25). All but one patient were admitted to the Department of Endocrinology CMHI before surgery. Informed consent was obtained from all patients who completed the questionnaire and Institutional Bioethical Commission (48/KBE/2018) gave permission for the release of anonymized data for publication.

### The latest follow-up

The latest FU of adult patients (previous patients of CMHI) was done using the data from the patient’s questionnaire (containing questions about the relapse of the disease, current medications and co-morbidities) completed by each patient. Answers to the questionnaire were obtained either by mail, telephone, or electronically and were verified with the patient by the doctor conducting the study. From 24 adult patients, 18 patients responded, so the response rate was 75%. FU of patients who did not respond to the questionnaire was finished during the last assessment at CMHI. In case of 1 patient there was no FU after TSS. The long-term FU of current CMHI patients was performed based on the data from the last clinical evaluation at CMHI and their/their parent’s consent was also obtained. In order to present the outcome confirmed by clinical evaluation, a long-term analysis of 27 patients (93%) was performed also on the basis of data from the latest clinical assessment at CMHI (long-term remission was reported as the need for glucocorticoid therapy or clinical or biochemical evidence of eucortisolism (defined as a morning serum cortisol from 5–25 μg/dl or normal 24-h urine cortisol or 17-OHCS excretion)).

### Hormone assays

Serum cortisol, triiodothyronine and thyroxine (free and total) were determined by immunochemical or radioimmunoassay method. Thyroid-stimulating hormone was measured by immunochemical or by radioimmunometric method. Serum ACTH, luteinizing hormone (LH) and follicle-stimulating hormone levels were determined by immunoradiometric assay and serum testosterone and estradiol—by radioimmunoassay. Cortisol levels in the morning and at night were defined as the mean values of 2 measurements respectively: cortisol level at 0800H and 0830H and cortisol level at 0000H and 0030H.

### Bilateral inferior petrosal sinus sampling

Bilateral inferior petrosal sinus sampling (BIPSS) was done by blood sampling from each inferior petrosal sinus for measurement of ACTH concentration simultaneously with peripheral venous sampling. ACTH was measured at baseline and at 3’, 5’, and 10’ after ovine corticotropin-releasing hormone (CRH) administration (1 μg/kg). Central ACTH secretion was confirmed by an inferior petrosal sinus (central) to peripheral ACTH ratio >2.0 at baseline and >3.0 after oCRH stimulation [18]. Lateralization of ACTH secretion was defined as an interpetrosal sinus ACTH gradient of >1.4 [19].

### Histopathological examination

Resected tissues were analyzed using hematoxylin and eosin staining, as well as reticulin staining and ACTH immunohistochemistry.

### Pituitary function

The pituitary function of adult patients was assessed at 2 time points: at the last clinical assessment at CMHI and at the latest FU, while the pituitary function of 4 children was assessed based on the information from the last visit at CMHI. At the latest FU adult patients gave information in the questionnaire about their drug therapy due to hormonal deficits since the definitive CD treatment. The evaluation of pituitary function at the last visit at CMHI was based on the following rules: secondary hypothyroidism was defined as a reduced (below the lower limit of the assay) level of free thyroid hormones with low, inappropriately normal or even slightly elevated (but inappropriate) thyroid-stimulating hormone concentration; secondary adrenal insufficiency was diagnosed on the basis of a reduced level of morning cortisol (<5 μg/dl) with low (most commonly) or inappropriately normal ACTH levels (ACTH reference range 10–60 pg/ml) and/or insufficient cortisol response in the exogenous ACTH test (an intravenous administration of 1 μg or 250 μg corticotropin), e.g. a cortisol level after stimulation below 18 μg/dL in the 30th or 60th minute of the test. Hypogonadotropic hypogonadism was diagnosed according to a lack of signs of puberty after the age of 13 in girls and 14 in boys accompanied by low levels of sex hormones (estradiol/testosterone) at low gonadotropin concentrations and/or based on an insufficient LH response in the LH-releasing hormone test (according to obligatory laboratory standard). The central diabetes insipidus (DI) was diagnosed on the basis of polyuria in patients with urine osmolality below 300 mOsm/kg with serum osmolality above 300 mOsm/kg or on the basis of a water deprivation test results. Short-term assessment of GH secretion was performed in accordance with current recommendations. The following stimulation tests were used to assess growth hormone secretion: with glucagon, with L-dopa, with clonidine and with arginine. GH deficiency (GHD) was defined as peak GH <10 ng/ml in 2 stimulation tests [20].

### Auxology and puberty staging

The obtained anthropometric parameters (initial and final) were presented as standard deviations (SDS) for body height and BMI using the LMS method [21,22]. Z-score values were calculated from the formula:

$$z-score(x)=\frac{{\left(\frac{X}{M}\right)}^{L}-1}{LxS},$$

The L, M, S values were taken from the tables of the reference system for a given age and sex[23, 24]. Adult height of previous CMHI patients was obtained from the patient’s questionnaire. Mid-parental target height (MPTH) was calculated according to the formula: (father's height + mother's height + 13 cm)/2 for boys and (father's height + mother's height—13 cm)/2 for girls [25]. Pubertal staging was assessed according to Tanner’s criteria [26] and pubertal stage at the latest FU was made at the last visit at CMHI.

### Statistical analysis

The distribution of continuous variables was checked for normality. The Student t-test was used to determine significant differences, when variables had the normal distribution. The Mann-Whitney test was used to compare groups with non-normal variable distribution. The exact Fischer test, one-proportion z-test and two-proportion z-test for small sample size were used to compare two groups where the checked variables were expressed in a dichotomous scale. Significance was assumed if P<0.05. Data were analyzed using Statistica 13.0 PL for Windows.

## Results

The mean age at the disease onset was 10.20 yrs. (the median age was 10.80 yrs., range 4.33–16.00). The disease onset was defined as the moment when the first symptom of the disease occurred–e.g.: time of weight gain or height deceleration at the growth chart and other. There was no significant difference between the patients regarding sex (P = 0.85). Detailed characteristics of anthropological parameters at presentation and at the latest FU are detailed in S1 Table. Mean height standard deviation score (SDS) for the whole group at presentation and at latest FU were respectively -1.71 (range 0.44 to -4.04) and -1.00 (range 1.01 to -3.36). Height SDS at FU had increased compared to the height SDS at presentation (P = 0.11). Regarding rGH treatment, the difference in height SDS at presentation and height SDS at FU was statistically significant in the group of patients treated with rGH (P<0,01). MPTH SDS for 23 patients with available data was -0.36 (range -1.74–0.75) and was different from the height SDS at presentation and height SDS at latest FU (P<0.05). The difference between MPTH and height SDS at latest FU (mean 0.79; range -0.89–2.69) was less than the difference between MPTH SDS and height SDS at presentation (1.53; range -0.56–3.38) (P<0.01). Mean body mass index (BMI) SDS at presentation was 1.36 (range 3.16 to -1.61) and 0.97 (range 1.55–0.41) at latest FU. BMI SDS was lower at latest FU (mean 0.97; range 1.55–0.41) compared to presentation mean BMI SDS 1.36 (range 3.16–1.61) but the difference was not significant (P = 0.18).

### Clinical features at diagnosis

**Fig 1:**
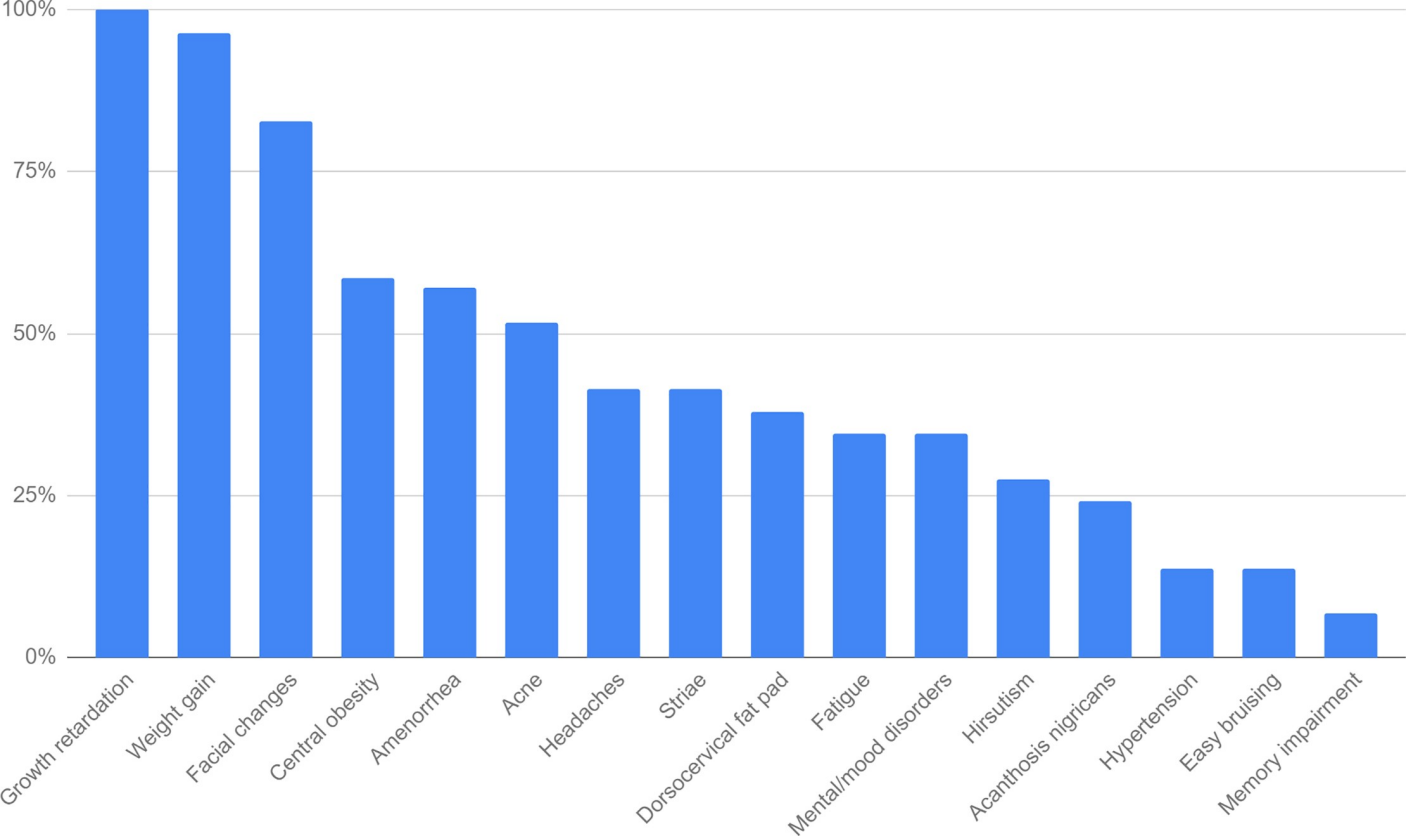
Clinical features at diagnosis of Cushing’s disease.

Mean age at diagnosis was 13.46 yrs. (range 5.50–17.30). The most common symptoms are presented in Fig 1. The most common features were: growth retardation (100%), weight gain (96%), facial changes: round face and facial plethora (83%). Other features: central obesity (59%), primary or secondary amenorrhea (57%), acne (52%), headaches (41%), striae (41%), dorsocervical fat pad (38%), fatigue (34%), mental and mood disorders: depression, anxiety and mood swings (34%) and hirsutism (28%) were also often diagnosed. Less common presenting features were: acanthosis nigricans (24%), hypertension (14%), easy bruising (14%) and memory impairment (7%). Mean symptoms duration prior to diagnosis was 3.26 yrs. (median 3.08; range 0.80–9.00). Testicular volume ≥4 ml was considered pubertal for boys and breast stage C2 or above for girls according to Tanner’s criteria [14, 15]. At presentation, 15 patients (55.6%), of which 5 (35.7%) were male, were pubertal and 12 patients (44.4%), of which 9 (64.3%) were male, were prepubertal. Data about pubertal stage of 2 patients are missing. All but one prepubertal patient (1 female) had signs of virilization with abnormally advanced pubic hair growth or genital development.

### Diagnostic investigations

#### Laboratory preoperative evaluation

Mean morning serum cortisol (at 0800H and 0830H) was 30.05 μg/dl (median 25.00, range 11.00–85.94). Mean sleeping serum cortisol (at 2400H and 2430H) was 21.67 μg/dl (range 8.20–52.14). Mean maximum morning serum ACTH (at 0800H) was 111.70 pg/ml (median 85.15; range 16.00–536.00; reference values 10–60). The following protocol of dexamethasone (Dx) administration was used: 30 μg/kg/dose (max. 0,5 mg/dose) every 6 hours for 48 h (low dose dexamethasone suppression test) and 120 μg/kg/dose (max. 2 mg/dose) every 6 hours for 48 h after low dose dexamethasone suppression test (high dose dexamethasone suppression test) [18]. In 18 patients (82%) from 22 with available data on the results of low dose dexamethasone suppression test cortisol failed to suppress <1.8 μg/dl after 48h. The data on the results of high dose dexamethasone suppression test was available in 27 patients (93%). Twenty-three out of twenty-seven patients (85%) showed suppression of cortisol to more than 50% of the basal pre-dexamethasone level. The corticotropin-releasing hormone stimulation test (CRH test) (1 μg/kg i.v.) was performed in 21 patients. An increase of serum cortisol ranged from 1.07% to 166.7% (median 50%), in 14/18 (77.8%) patients the cortisol rise was >20% from baseline. In 3 patients there was no cortisol rise after CRH. The difference of serum ACTH level between the mean value 15’ and 30’ after CRH stimulation and baseline ranged from -44% to 644.9% (median 95.7%), in 14/21 (66.7%) patients the ACTH rise was >35% from baseline. In 2 patients (9.5%) there was no ACTH increase after ACTH stimulation. The summary of diagnostic studies is presented in Table 1.

**Table 1:** Diagnostic studies.

Diagnostic studies	No.	%
Absent diurnal cortisol rhythm^a^	29/29	100
Failure to cortisol suppression by low dose dexamethasone	18/22	82
Cortisol suppression by high dose dexamethasone^b^	23/27	85
High^c^ 24-h urine free cortisol	21/23	91.3

^a^Means of 2 serum cortisol values obtained at 0730–0800H and at 0000–0030H

The presence of diurnal cortisol rhythm defined when serum cortisol concentration reached its zenith in the morning (06.00–08.00 h) and its nadir in the night during the first half of normal sleep [2]

^b^Cortisol reduced from baseline by ≥50%

^c^More than 80 μg/m2 [2]

#### Imaging

Magnetic resonance imaging (MRI) of the hypothalamo-pituitary region showed abnormalities consistent with a pituitary adenoma in 20/29 cases (69%). Concordance between the site of the adenoma in MRI and at surgery was 9/17 (53%) (not all data of adenoma position at TSS available). Localization of a tumor in preoperative imaging raised the cure rate for CD to 70% when MRI detected pituitary tumor vs. 30% when there was no tumor in MRI, but the difference was not significant (P = 0.68).

#### Bilateral inferior petrosal sinus sampling

Thirteen out of twenty-nine patients (45%) who had negative preoperative MRI or unclear endocrine diagnosis underwent BIPSS, which was performed with general anesthesia in all patients. The mean age of patients at the time of BIPSS was 11.70 yrs. (range 5.17–17.00). Positive BIPSS results were found in all 13 patients (100%). BIPSS showed lateralization of ACTH secretion in 8 patients (62%; 6 right, 2 left). There were no data about lateralization in BIPSS in Patient 8’s medical record, in case of Patient 19 the BIPSS results were ambiguous, in case of Patient 26 BIPSS gave no results because of abnormal venous system (collateral circulation). Concordance between lateralization of the tumor on BIPSS and during surgery was 5/8 (63%). One out of five patients (20%) with this concordance did not have remission after TSS1. The remission rate (after TSS1) in patients who underwent BIPSS preoperatively was 84.5% (11/13 patients) and 80% in patients who did not undergo this procedure (12/15 patients); (1 patients excluded because of no FU); the difference was not statistically significant (P>0.5). Localization of a microadenoma by BIPSS agreed with surgical location in 62.5% of cases. The results of the imaging investigations are shown in Table 2.

**Table 2:** Characteristics of pre-TSS diagnostics and the long-term outcome of each patient.

Pat. No.	Adenoma position in MRI	BIPSS	Adenoma position at TSS	Histology	Definitive treatment	Time of HC substitution from definitive treatment	Rec. post definitive treatment (yrs.)	Long-term outcome
1	R	-	No data	Adenoma	TSS + (BA after rec.)	From TSS1 to Rec (1 yr); after BA to now (9 yrs.)	Yes (1.00)	Remission; Nelson’s syndrome (1.50 yrs. post BA)
2	ML*	-	L	Adenoma	TSS1+TSS2	From TSS2 to latest FU (10.08 yrs.)	No	Remission
3	L	R	R	Adenoma	TSS1	0.83 yrs.	No	Remission
4	N	L	L	Adenoma	TSS1	Until latest FU (13.50 yrs.)	No	Remission
5	R*	-	R	Adenoma	TSS1	2.00 yrs.	No	Remission
6	R*	-	R	Adenoma	TSS1	Without HC	Rec suspicion	Rec. suspicion at latest FU
7	ML	ML	L	Adenoma	TSS1	Until latest FU (11.80 yrs.)	No	Remission
8	R**	Yes ***	No data***	Adenoma	TSS1+TSS2+XRT	From XRT to latest FU (23.83 yrs.)	No	Remission
9	L	ML	L	Adenoma	TSS1	Until latest FU (9.50 yrs.)	No	Remission
10	N	-	No data***	No data***	TSS1	0.80 yrs.	No	Remission
11	L	-	No data	Adenoma	TSS1	2.17 yrs.	No	Remission
12	R*	-	R	Adenoma	TSS1	Until latest FU (16.17 yrs.)	No	Remission
13	ML*	-	ML	Adenoma	TSS1	Until latest FU (17.58 yrs.)	No	Remission
14	N	L^a^	No data***	No data***	TSS1	3.25 yrs.	No	Remission
15	L*	-	L	Adenoma	TSS1	0.33	Rec. suspicion	Rec. suspicion
16	ML*	-	R	Adenoma	TSS1	0.92	No	Remission
17	N	-	No data***	No data***	TSS1	3.25	No	Remission
18	R	-	ML	Adenoma	TSS1+TSS2	No HC substitution	No	Remission
19	N (EPG)	+/-	R/ML	Adenoma	TSS1	Until latest FU (7.92 yrs.)	No	Remission
20	N	R*	R	Adenoma	TSS1+TSS2+XRT+BA after Rec	4.40 yrs. from XRT to Rec., after BA on HC	Yes (3.70 from XRT)	Remission
21	L	R	ML	Adenoma	TSS1+TSS2	1.75 from TSS1 to Rec.	Yes (1.75 yrs. from TSS1)	Death 1 month after TSS2
22	ML*	-	No data	Adenoma	TSS1+TSS2*+BA (4 times)	8.00 yrs. from TSS1 to Rec.	Yes (8.08 yrs. from TSS1)	Remission
23	N (NH FL)	-	No data***	No data***	-	-	-	Persistent disease (after TSS1 and XRT)
24	L*	-	No data	Adenoma	TSS1	Until latest FU (24.33 yrs.)	No	Remission
25	L*	-	L	Adenoma	No data	No data	No data	No data
26	ML	****	ML	Adenoma	TSS1	Until latest FU (8.33 yrs.)	No	Remission
27	N	R	No data	Focal corticotroph cells hyperplasia	TSS1	Until latest FU (2.33 yrs.)	No	Remission
28	N (NH FPL)	R	R/ML	No adenoma (normal pituitary gland)	TSS1	0.50 yr	No	Remission
29	R**	R	R	Adenoma	TSS1	Until latest FU (0.67 yrs.)	No	Remission

BIPSS—bilateral inferior petrosal sinus sampling

(-)—not performed

L—left-sided lateralization/adenoma position, R—right-sided lateralization/adenoma position; ML—no lateralization/ midline position

MRI—magnetic resonance imaging, N-normal imaging (no adenoma), EPG-enlarged pituitary gland, NH FL—non-homogeneous front lobe, NH FPL—non-homogeneous frontal and posterior lobe

*- outside CMHI

**- adenoma detected in the 2nd MRI

***- outside CMHI

****-unsuccessful—abnormal venous system—collateral vein of the right femoral vein and left jugular vein

+/-—ambiguous result

^a^ but no data about lateralization

#### Transsphenoidal pituitary surgery

Treatment of CD consisted of TSS as the first definitive therapy in all patients. Regarding surgeries performed at CMHI, an endoscopic technique was used only in individual cases due to the weak sinuses aeration in prepubertal children, hence the main technique was transnasal microsurgical transsphenoidal resection. Twenty-three out of twenty-eight patients (83%) were operated at CMHI by the same neurosurgeon. Twenty-three out of twenty-eight patients (83%) had the initial biochemical remission after a single TSS procedure (post-operative serum cortisol <1.8 μg/dl [27]) and 2/28 (7%) patients after two consecutive TSS procedures (Patient 2 and 18). The overall rate of remission following TSS was 89%. One patient was not included into this analysis because of no FU after TSS. The remission rate after TSS1 performed by the same neurosurgeon from CMHI was 83.3% (20/24 patients), after TSS2 8.33% (2/24 patients) and in total 91.67%. In all patients (with available data) a pituitary microadenoma was identified at TSS (Table 2).

#### Pituitary histology

Histopathological examination confirmed a corticotroph adenoma in 23/25 (92%) patients. In 4 patients (Nos. 10, 14, 17 and 23) operated outside CMHI there were no data in the medical record about the result of histopathological examination. Positive histology (pituitary adenoma) was present in all 19/19 patients (patients with available data) with remission after TSS1 and in 4/4 patients without remission (Table 2). Histopathological examinations of Patients 27 and 28 revealed focal corticotroph hyperplasia and normal pituitary gland, respectively. Both patients were in remission at latest FU.

#### Pituitary Irradiation

Two patients (7%) (Patients 8 and 20) had persisting postoperative hypercortisolemia after two TSS and received external beam direct pituitary irradiation outside our center. Pituitary irradiation in patient 20 was performed using a 6-MV linear accelerator and a dose of 4.500 cGy (rad) in 25 fractions was delivered every weekday for 5 weeks. There were no detailed data on XRT in Patient 8’s medical record.

### Long-term outcome

At latest clinical assessment at CMHI, 77.8% of patients were in remission, 2 patients (7.4%) had recurrence of disease, while 1 patient (3.7%) had the suspicion of recurrence. One patient (3.7%) died after TSS2. Two patients (7.4%) were before TSS. The median period of FU from TSS for all patients except two (Nos. 12 and 25) from TSS was 3.87 yrs. (range 0.17–10.17).

**Fig 2:**
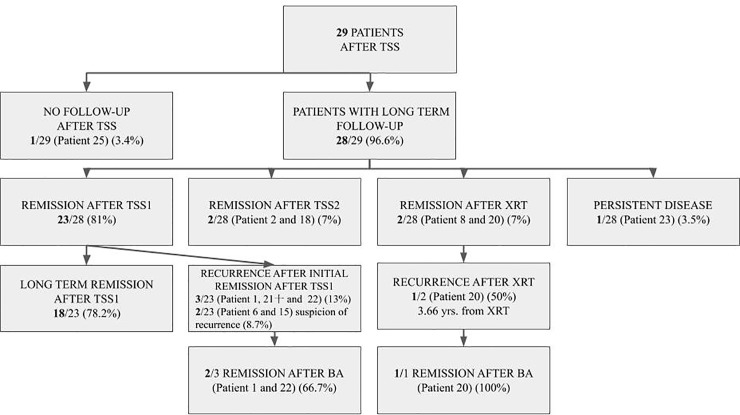
The summary of latest follow-up of analyzed patients.

At the moment of the end of FU: 86% of patients were in remission, 2 patients (7%) had the suspicion of recurrence, 1 patient (3.5%) was uncured, 1 patient (3.5%) died after TSS. Three patients underwent bilateral adrenalectomy. The median FU from TSS for all patients except one (No. 25) was 10.23 yrs. (range 0.67–24.50). Fig 2 shows the summary of latest FU (based on self-reported data from 64% of patients and data from medical records from 36% of patients) (Fig 2).

#### Recurrence rates and management

Clinical and biochemical recurrence of hypercortisolemia was seen in 4 (15%) patients (Patients 1, 20, 21 and 22) (Table 2). The clinical features in Patient 1 were: weight gain, headaches and malaise. There was no evidence of adenoma in MRI, ketoconazole therapy gave no result in lowering cortisol level. The patient underwent 2-stage bilateral adrenalectomy (12 and 13 yrs. after successful TSS). 1.5 yrs. after the second stage of BA, Nelson’s syndrome occurred and the patient underwent sella turcica exploration. The clinical feature of recurrence in Patient 20 was excessive body weight 3.50 yrs. after XRT (his second TSS was unsuccessful and biochemical remission was achieved after XRT). Pharmacotherapy with ketoconazole did not lower the serum cortisol level and the patient achieved remission after BA. Patient 21 presented weight gain (5 kg/5 months) and height deterioration 1.75 yrs. after successful TSS. Total hypophysectomy was performed, but after the procedure ischemic stroke was diagnosed, which caused brain herniation and death 1 month after TSS2. Patient 22 suffered from malaise, mood change and weight gain 8.08 yrs. after successful TSS. Furthermore dorsocervical fat pad, red stretch marks and hypertension were diagnosed. He underwent unsuccessful pituitary exploration and 1 unsuccessful BA. The next surgeries—two-stage bilateral readrenalectomies gave final remission. Mean recurrence time from initial remission in four patients was 3.62 yrs. (range 0.92–8.08).

#### Time of recovery of pituitary adrenal axis post-TSS or RT

Twenty-six out of twenty-nine (90%) patients required hydrocortisone (HC) substitution after definitive treatment (TSS1/TSS2 or XRT). Fifty nine percent of patients were on HC substitution at the time of the final FU. Time of recovery of pituitary adrenal axis post-TSS in patients who required HC substitution (8 patients) after successful treatment was 1.66 yrs. (range 0.33–3.25).

#### Anterior and posterior pituitary function: GH secretion—Short-term assessment

Short-term growth hormone (GH) assessment was performed in 12 patients at a mean interval of 1 year (range 0.08–8.33) after the last definitive treatment for CD which was TSS for all patients (mean peak GH 4.93 mIU/l, range 0.11–36.8). From other patients: 1 patient (No. 14) had been treated with recombinant growth hormone (rGH) before TSS and the treatment was continued after surgery, 1 patient (No. 26) was undergoing evaluation for GHD at the last assessment, in 1 patient GH assessment has been performed before definitive treatment (XRT). GHD was present in 11/12 (92%) tested patients (mean peak GH 1.90 mIU/l, range 0.11–7.80). In total, rGH therapy was administered in 13/15 patients (86.7% of all tested). In 3 patients rGH therapy was discontinued because of the disease recurrence. 1 patient (No. 3) was before start of rGH therapy at the last assessment. At latest assessment none of the surveyed patients were treated with rGH (there is no reimbursement for rGH therapy in Poland after growth completion). The rGH therapy was finished at the mean age 17.69 yrs. (range 10.83–22.40).

#### Anterior and posterior pituitary function: Evaluation of development of sexual features

On the basis of available data from the last visit at CMHI, 19/25 patients (12 males) were undergoing pubertal development and 5/25 patients (2 males) were pubertally adults (S2 Table). One patient did not start pubertal development at latest evaluation (Patient 27, age at the moment of latest evaluation 12.83 yrs.). Pubertal induction has been performed in 11 patients (Patients 2, 4, 7, 8, 9, 12, 13, 18, 20, 22, 24). At latest FU 12 patients (Patients 1, 4, 7, 8, 9, 12, 13, 15, 18, 20, 22, 24) were taking sex hormone therapy (10 of them (83.3%) had pubertal induction before). Five patients (4 females) reported to have children and 4 of them (3 females) were on sex hormonal therapy at latest FU. Three females reported problems with getting pregnant (Patients 9, 12, 13), however each of them has one child. One woman reported that in vitro fertilization is planned in her case.

#### Anterior and posterior pituitary function: Central diabetes insipidus

Eighteen out of twenty-nine patients (62%) developed central DI after TSS1. The mean time of DI duration after TSS1 was 38.40 months (range 0.03–287.00). Normal production of antidiuretic hormone recovered in 13/18 patients (72.2%) with a mean time 5.76 months (range 0.03–19.00). At latest evaluation 4 patients (Patients 9, 15, 19, 22) were taking desmopressin as vasopressin substitution therapy. In 1 patient (Patient 11) DI lasted 6 days, after that symptoms of syndrome of inappropriate antidiuretic hormone hypersecretion occurred, which disappeared after fluid restriction.

#### Long-term pituitary function assessment

The evaluation of the long-term pituitary function was performed in 27 out of 29 (93%) patients. Patient 21 was excluded from the analysis because post-TSS2 complications resulted in the patient’s death 1 months after surgery. Patient 25 was referred to post-TSS care to her place of residence and did not respond to the questionnaire. The mean FU of patients included to the analysis was 10.52 yrs. (range 0.67–24.50). At the time of the last clinical assessment at CMHI pituitary thyroid-stimulating hormone deficiency was diagnosed in 73.9% of patients, adrenocorticotropin deficiency in 56.2% of patients, gonadotropin deficiency in 43.4% and antidiuretic hormone deficiency in 21.7% of patients. At the time of the last FU, 63% of patients were on levothyroxine therapy since definitive treatment, 59% of patients were on hydrocortisone treatment, 37% of patients were taking sex hormones replacement, 15% were taking antidiuretic hormone. At latest FU, 6 patients (22%) had a single pituitary hormone deficiency. Seven patients did not take any substitution therapy for pituitary deficiency.

#### Co-morbidities: Hypertension

At diagnosis, 5 (17%) patients (Patients 5, 17, 22, 23, 29) had hypertension, which resolved in all of them (the mean time of FU of 7.23 yrs. (range 0.67–22.83)). Patient 16, who did not suffer from high blood pressure at diagnosis, reported at latest FU to take ramipril due to primary hypertension.

#### Co-morbidities: Cognitive disorders and depression

At diagnosis

At diagnosis, low mood was found in 3 (10%) patients (Patients 1, 9 and 23) and emotional lability in 6 (20%) patients (Nos. 7, 20, 26, 27, 28 and 29). Patient 1 developed clinical depression diagnosed before TSS and he was treated with fluoxetine. Two patients (Nos. 10 and 11) had problems with concentration. Patient 6 presented very strong anxiety, a sense of threat of disease and distrust immediately after TSS. From the day 3 after TSS she had visual and tactile hallucinations. She was taking chlorprothixene for 2 weeks and the psychotic episode resolved (resolution of the hypercortisolemia was observed 1 month later).

Follow-up

Patients 10 and 12 reported in the questionnaire having depression and being under constant psychiatry care (FU 6 and 16.17 yrs.) but they do not require anti-depressant therapy. None of these two patients had any psychiatric problems before TSS. Thirty-eight percent of 18 surveyed adults reported low mood, 33%—depressive states, 44%—memory problems, 50%—emotional lability, 39%—problems with concentration, 61%—mood swings and 67%—irritability.

## Discussion

This research supports previous findings on clinical presentation of CD in the pediatric population, however, it also indicates different aspects of the disease in comparison with other studies. Comparing to studies on adult population, there were identified both similarities and differences in clinical presentation of CD. Similar to the results of the study by Devoe et al. on 41 children [7] and the study by Knappe et al. on 55 children [28], but contrary to other results on pediatric population reporting a male predominance [6, 15] there was no sex domination in the analyzed group. This result is in contrast to the results on adult population with female predominance [6, 11, 12]. Mean symptoms duration prior to diagnosis was 3.26 yrs. (the median 3.08; range 0.80–9.00), which is relatively long period in comparison with results presented in the pediatric literature: from 1.8 yrs. (range 0.50–3.50) in Yordanova et al.’ study (21 children with CD) [15], 2.33 yrs. (range 0.25–7.00) in Batista et al.’ study (72 children with CD) [29], 2.5 ± 1.7 yrs. (range 0.30–6.60) in Savage et al.’ study (37 children with CD) [16] to 3± 2 yrs., (range 3 mos.-7 yrs.) in Magiakou et al.’ study (50 children with CD) [30]. However, the mean duration of symptoms in our study is shorter than the time reported in large studies on adults (4 yrs.) [11, 12]. Suppression of linear growth and weight gain were the most common features in our patients, which is in accordance with other pediatric studies [6, 7, 8, 15, 16]. Regarding symptoms commonly seen in adults with CD [31], central obesity and moon facies were often reported in our pediatric group, but hypertension and diabetes were not often seen in this group.

On the basis of data on adult patients, the localization of a tumor in preoperative MRI raises the cure rate of CD: up to 90% (if MRI detects pituitary tumor) vs. 50–70%, when there is no tumor in MRI [12, 32, 33]. In the presented study the results are similar to those presented in adults and indicate that MRI plays an important role in CD treatment process. However, in our study the difference was not statistically significant (P = 0,68). In our analysis the detection rate of pituitary adenoma in MRI was 69% and this result is similar to other studies on the groups from 21 to 72 children, where tumor visualization was in 43–72% [6, 7, 15,16] and the result is also similar to adenoma detection rates in adults: 50–70% [34, 35, 36]. The accuracy of BIPSS in prediction of adenoma site at surgery has been checked in several studies [37, 38, 39, 40]. The percentage of predictive lateralization was from 58% in Batista et al.’ study (94 children) [32], 81% in Storr et al.’ (41 children) [33], 91% in Lienhardt et al.’ analysis on 17 children [34] and also 91% in Dias et al.’ study (17 children) [35] to even 100% in Storr et al.’ study (but only 4 cases) [41]. In our study localization of a microadenoma by BIPSS agreed with surgical location in 62,5% of cases which puts into question the usefulness of BIPSS in adenoma localization. The fact that BIPSS is not reliable enough for adenoma localization was confirmed also in the publication from 2018 where BIPSS localizes adenoma in only 54% of CD pediatric patients [42]. Furthermore, relatively low percentage of microadenoma localization in BIPSS and during surgery may result from changes of ACTH levels during general anesthesia, as the increase of ACTH values during surgery were noted among respondents under general anesthesia in other types of invasive procedures [43]. However, the initial remission rate in patients who underwent BIPSS before TSS was 84.5% and 80% in those without this procedure before surgery, which is similar to the results in Storr et al.’ analysis [6].

The remission rates in pediatric population vary largely due to the lack of agreement on definition of post-operative remission, with reported rates between 45 and 95% [4, 5, 6, 7, 8, 9]. They depend also on the neurosurgeon’s experience and time of FU. The initial remission rate (82% after TSS1 and 89% in total) in the presented group compares favorably with other published results. The recurrence rate in children is reported to be between 2 and 27% after initial remission [4, 6, 8, 44], which is similar with the rate of adult patients: recurrence rate between 20 and 30% during the first 5 yrs. following definitive treatment [45, 46]. In our study the recurrence rate was 15%, however 2 patients had suspicion of CD recurrence at the end of FU, so the rate could be even higher than presented (maximum 21%). The longest period from the definitive treatment to the relapse was 8.08 yrs., which emphasizes the need for long-term care for patients after CD treatment and increased vigilance after many years of treatment. Relatively high recurrence rate confirms that the longer time of FU, the higher rate of relapse is. Interesting to note is the percentage of patients on HC substitution at latest FU, which was higher in comparison with the moment of the last evaluation at CMHI (at the time of the last clinical assessment at CMHI 56.2% of patients were on HC substitution, while at the time of the last FU 59% of patients). This difference may result from the definitive CD treatment–there were 3 patients after BA, which results in chronic HC substitution.

The limitation of the data obtained from the patient’s questionnaire is the fact that the pituitary function was not assessed on the basis of the results of hormonal tests. However, this information is valuable and the knowledge about pituitary function after a long period from TSS is important both for patients and for doctors, while limited number of pediatric studies report this kind of data. However, the FU based on biochemical evidence (at latest clinical assessment) was shorter but still long-term (mean observation time 3,87 yrs.). High percentages of patients on ongoing levothyroxine therapies in the presented study may result from the fact that other thyroid disorders may be a reason for needing drug supplementation. In each patient levothyroxine therapy lasted from start of drug therapy (after TSS) until latest FU, which indirectly suggests the persistence of the pituitary deficiency, although there is a possibility of a different cause than the central one. The patients were asked in the questionnaire about other chronic diseases and none of them gave the primary thyroid disease as the potential reason of hypothyroidism. These results are a bit controversial as in other studies on patients after TSS in childhood the thyroid-stimulating hormone deficiency was reported to be between 6.7% [7] and 9.5% [15]. Obtained results (laboratory confirmed secondary hypothyroidism in 73.9% of patients at the time of the last clinical evaluation, as well as levothyroxine supplementation at latest FU in 63% of patients) are much higher in comparison with other studies in which the thyrotropin deficiency in patients after TSS in childhood was reported to be between 6.7% [7] and 9.5% [15]. The relatively high rates of hormone deficiencies on long-term FU compared to other series may be caused by more extensive pituitary surgery resulting in a high remission rate. In addition, the material comes from a long period of time when imaging, accessibility of diagnostic methods (e.g. BIPSS) and surgical technique were not ideal.

Most patients did not have the evaluation of GH secretion at latest FU so our results cannot be comparable with for example Devoe et al.’ or Yordanova et al.’ results, where GHD was the most common pituitary deficiency [7, 15]. Despite the limitations of this study, data obtained from the questionnaire gave important information about patients’ functioning in a long period after CD treatment, which advances other studies lacking the long-term follow-up perspective.

## Conclusions

CD recurrence can occur even a long time after effective treatment. Long-term observation of patients after CD treatment in childhood indicates the presence of a large number of patients with hormonal pituitary deficits, as well as a significant problem of mood and cognitive disorders that may be the result of hypercortisolemia in the past.

## Supporting information

S1 Table Characteristics of the patients at presentation and at latest follow-up. (DOCX)

S2 Table Puberty features. (DOCX)

S1 Appendix Questionnaire for adult patient. (PDF)

## Abbreviations

ACTH: adrenocorticotropic hormone
BA: bilateral adrenalectomy
BMI: body mass index
BIPSS: bilateral inferior petrosal sinus sampling
CD: Cushing’s disease
CMHI: Children’s Memorial Health Institute
CRH: corticotropin-releasing hormone
DI: diabetes insipidus
FU: follow-up
GH: growth hormone
GHD: growth hormone deficiency
HC: hydrocortisone
MPTH: mid-parental target height
MRI: magnetic resonance imaging
LH: luteinizing hormone
Pat.: patient
Rec.: recurrence
rGH: recombinant growth hormone
SDS: standard deviation score
TSS: transsphenoidal pituitary surgery
TSS1: 1^st^ transsphenoidal pituitary surgery
TSS2: 2^nd^ transsphenoidal pituitary surgery
XRT: radiotherapy
yrs: years
