# Supplementary material for: Long-term outcome in patients after treatment for Cushing’s disease in childhood
Source: PLoS One. 2019 Dec 12;14(12):e0226033. doi: 10.1371/journal.pone.0226033 (PMC6907843; doi:10.1371/journal.pone.0226033)
Supplement: S1 Table — (DOCX) [file pone.0226033.s001.DOCX]

**S1 Table. Characteristics of the patients at presentation and at latest follow-up.**

Puberty staged by Tanner [^[[1]](#endnote-1)^]. B - breast development, G - genital stage, P - pubic hair stage, A - axillary hair stage, M - menarche, M1 - menarche occurred, M0 - no menarche, TV - testicular volume (ml), R - right, L - left, FU - follow up

|  | | **At presentation** | | | | **FU**  **[yrs.]** | **At latest follow-up** | | | |
| --- | --- | --- | --- | --- | --- | --- | --- | --- | --- | --- |
| **Pt no.** | **Sex** | **Age [yrs.]** | **Height SDS** | **BMI SDS** | **Pubertal stage (TV: R/L)** |  | **Age [yrs.]** | **Height SDS** | **BMI SDS** | **Pubertal stage (TV: R/L) [age at final clinical assessment [yrs.]]** |
| 1 | M | 17 | -0.84 | 1.95 | A3 G3 P4 (3/4) | 10.67 | 28 | 0.68 | 0.93 | A3 G3 P4 (6-8/6-8) [17] |
| 2 | M | 15 | -1.50 | 1.78 | A3 G2 P4 (2/2) | 10.08 | 25 | -2.14 | 1.08 | A3 G4 P4 (6-8/6-8) [18] |
| 3 | M | 16 | -1.82 | -0.49 | A4 G4 P4 (20/20) | 0.83 | 18 | -2.25 | 1.23 | A4 G4 P4 (20/20) [18] |
| 4 | F | 11 | -0.72 | 1.76 | A2 B2 P3 M0 | 13.50 | 18 | 0.06 | 1.12 | A2,B3 P3 M1 (after hormone therapy) [18] |
| 5 | M | 14 | -2.18 | 1.26 | A3 G3 P4 (7/7) | 1.83 | 15 | -0.96 | 1.06 | Adult [15] |
| 6 | F | 17 | -2.65 | 1.84 | A3 B4 P4, M1 [12 yrs.] | 1.17 | 18 | -3.06 | 0.59 | Secondary amenorrhea (no other data available) [18] |
| 7 | M | 15 | -3.07 | 2.33 | A3, G3 P4 (6/6) | 11.08 | 27 | -1.05 | 0.69 | A3 G4 P4 (8/8-10) [19] |
| 8 | F | 15 | -4.04 | -1.12 | A2 B2 P3 M0 | 24.50 | 40 | -0.86 | 0.93 | A2 B4 P2 M1 [26] |
| 9 | F | 12 | -0.34 | 1.20 | A2 B1 P3 M0 | 9.50 | 22 | 0.67 | 1.21 | A1 B3/4 P3 M1) [18] |
| 10 | M | 14 | -1.08 | 2.26 | A4 G4 P4 (15-18/15-18) | 6.00 | 21 | -1.20 | 0.90 | Adult (24/24) [17] |
| 11 | F | 13 | -0.97 | 0.37 | A4 B4 P4 M1 [12 yrs.] | 6.75 | 20 | -1.19 | 1.25 | Adult [18] |
| 12 | F | 15 | -0.62 | 3.11 | No data | 16.17 | 32 | 1.01 | 1.06 | No data |
| 13 | F | 15 | -3.21 | 0.83 | A2 B3 P4 M0 | 17.58 | 33 | -1.89 | 0.97 | Adult [21] |
| 14 | F | 12 | -3.78 | -1.61 | A1 B2 P3/4 M0 | 14.50 | 29 | -1.70 | 1.06 | A3 B4 P4 M1 [18] |
| 15 | F | 16 | 0.44 | -0.30 | A2 B3 P4 M1 [15 yrs.] | 14.75 | 31 | 0.84 | 0.83 | A4 B4 P4 M1 [18] |
| 16 | F | 17 | -0.60 | -0.15 | A4 B5 P5 | 16.67 | 34 | -0.89 | 0.87 | Adult [18] |
| 17 | F | 13 | -2.30 | 1.41 | A1 B3 P3 M0 | 4.42 | 18 | -0.63 | 1.17 | No data |
| 18 | M | 10 | -2.41 | 0.84 | A1 G1 P3 (1-2/1-2) | 7.92 | 19 | -1.14 | 1.13 | A5 G4 P5 (6/6) [19] |
| 19 | M | 17 | -3.64 | 1.89 | A4 G4 P4 (13-14/13-14) | 14.17 | 32 | -1.83 | 0.85 | A4 G4 P4 (25/25) [19] |
| 20 | M | 11 | -1.79 | 1.82 | A1 G2 P2 (2/2) | 15.58 | 27 | -0.58 | 0.82 | A2 G4 P4 (3-4/3-4) [17] |
| 21 | F | 9 | -2.47 | 2.39 | A1 B1 P2 M0 | 2.50 | 12 | -3.36 | 0.79 | A2 B2 P3 M0 [12] |
| 22 | M | 12 | -0.54 | 1.51 | A2 G2 P4 (3/3) | 22.83 | 35 | -0.58 | 0.71 | A3 G4 P3 (8-10/8-10) [21] |
| 23 | F | 16 | -1.40 | 3.16 | A3 B2 P5 M1 [14 yrs., later secondary amenorrhea diagnosed] | 5.42 | 23 | -1.53 | 0.41 | No data |
| 24 | M | 13 | -2.25 | 1.18 | A1 G2 P4 (2-3/2-3) | 24.33 | 38 | -0.42 | 1.00 | A5 G4/5 P4/5 (5/5) [20] |
| 25 | F | 10 | -1.63 | 3.05 | No data |  | No FU | No data |  | No data |
| 26 | M | 5 | -1.37 | 1.95 | A2 G2 P2 (3/3) | 8.33 | 14 | -1.05 | 1.55 | A1G2/3 P2 (8/8) [14] |
| 27 | M | 10 | -0.23 | 1.73 | A1 G1 P2/3 (2/2) | 2.33 | 13 | 0.01 | 0.61 | A1 G1 P1 (2/2) [13] |
| 28 | M | 11 | -0.87 | 1.07 | A1 G1 P2/3 (3/3) | 1.42 | 13 | -1.12 | 1.46 | A2 G2 P3 (6/6) [13] |
| 29 | F | 7 | -1.58 | 2.48 | A1 B1 P1 M0 | 0.67 | 8 | -1.73 | 0.77 | A1 B1 P1 M0 [8] |

1. . Falkner B, Daniels SR. Summary of the fourth report on the diagnosis, evaluation, and treatment of high blood pressure in children and adolescents. Hypertension. 2004;44(4):387–388. [↑](#endnote-ref-1)
