# Supplementary material for: Long-term outcome in patients after treatment for Cushing’s disease in childhood
Source: PLoS One. 2019 Dec 12;14(12):e0226033. doi: 10.1371/journal.pone.0226033 (PMC6907843; doi:10.1371/journal.pone.0226033)
Supplement: S2 Table — (DOCX) [file pone.0226033.s002.DOCX]

**Supporting information**

**S2 Table. Puberty features**

| **Pat. No.** | **Sex** | **Pubertal stage at last visit at CMHI (TV: R/L [ml]) [age at final clinical assessment [yrs.]]** | **Puberty features** | **Pubertal induction** | **Hormonal sex therapy at latest FU [age in yrs.]** | **Having children at latest FU [number of children]** |
| --- | --- | --- | --- | --- | --- | --- |
| 1 | M | A3 G3 P4 (6-8/6-8) [17] |  | No | Oral DHEA [28] | Yes [1] |
| 2 | M | A3 G4 P4 (6-8/6-8) [18] | HH | Yes (Testosterone +DHEA) | No [26] | No |
| 3 | M | A4 G4 P4 (20/20) [18] |  | No | No [18] | No |
| 4 | F | A2 B3 P3 M1 [18] | Menarche after induction | Yes (oral estrogen and progestagen) | Oral estrogen and progestagen [24] | No |
| 5 | M | Adult [15] |  | No | No [16] | No |
| 6 | F | A3 B4 P4 M1^a^ [17] | ^a^ | No | No [18] | No |
| 7 | M | A3 G4 P4 (8/8-10) [19] | HH | Yes (Testosterone) | Testosterone [27] | No |
| 8 | F | A2 B4 P2 M1[26] | HH | Yes (Oral estrogen and progestagen) | Oral Estradiolum, Norethisteroni acetas [40] | Yes [1] |
| 9 | F | A1 B4 P3 M1 [18] | HH | Yes (Oral estrogen, DHEA then oral estrogen + norgestrel) | Oral estrogen+dydrogesterone [22] | No |
| 10 | M | Adult (24/24) [17] | - | No | No [21] | No |
| 11 | F | Adult [18] | Dydrogesterone for 1 year due to secondary amenorrhea 3 yrs. after TSS | No | No [20] | No |
| 12 | F | No data | No data | Yes (oral estrogen and progestagen) | Oral Estradiolum, Norethisteroni acetas [32] | No |
| 13 | F | Adult [21] |  | Yes (Oral estrogen+norgestrel until latest FU) | Oral Estradiolum, Norethisteroni acetas [33] | Yes [1] |
| 14 | F | A3 B4 P4 M1 [18] | Tryptoreline for 1.25 yrs. | No | No [30] | No |
| 15 | F | A4 B4 P4 M1[18] |  | No | Oral Estradiolum, Norethisteroni acetas [40] | Yes [3] |
| 16 | F | Adult [18] | Secondary amenorhea for 10 months, menorrhea occurred 7 months after TSS | No | No [34] | Yes [2] |
| 17 | F | No data |  | No | No [18] | No |
| 18 | M | A5 G4 P5 (6/6) [19] |  | Yes (HCG, then testosterone) | Testosterone [19] | No |
| 19 | M | A4 G4 P4 (25/25) [19] |  | No | No [32] | No |
| 20 | M | A2 G4 P4 (3-4/3-4) [17] |  | Yes (Testosterone) | Menotropine +Choriogonadotropin alfa [27] | No |
| 21 | F | A2 B2 P3 M0 [12] |  | No | No [11] | No |
| 22 | M | A3 G4 P3 (8-10/8-10) [21] |  | Yes (HCG, then testosterone) | Testosterone [35] | No |
| 23 | F | No data |  | No | No [22] | No |
| 24 | M | A5 G4/5 P4/5 (5/5) [20] |  | Yes(HCG+Testosterone+Danazol) | Testosterone [38] | No |
| 25 | F | No data |  | No data | No data | No data |
| 26 | M | A1G2/3 P2 (8/8) [14] |  | No | No [14] | No |
| 27 | M | A1 G1 P1 (2/2) [13] |  | No | No [13] | No |
| 28 | M | A2 G2 P3 (6/6) [13] |  | No | No [13] | No |
| 29 | F | A1 B1 P1 M0 [8] |  | No | No [8] | No |

^a^secondary amenorrhea at latest FU (last menstruation before TSS (age 17 yrs.), a normal response in LH-RH test performed at the age of 18 yrs.
