# Supplementary material for: Long-term outcome in patients after treatment for Cushing’s disease in childhood
Source: PLoS One. 2019 Dec 12;14(12):e0226033. doi: 10.1371/journal.pone.0226033 (PMC6907843; doi:10.1371/journal.pone.0226033)
Supplement: S1 Appendix — (PDF) [file pone.0226033.s003.pdf]

Questionnaire for adult Patient

1. First name
   …………………………………………………………………………………………
2. Surname
   ………………………………………………………………………………….....
3. Date of birth
   ………………………………………………………………………….………..
4. Telephone number
   ………………………………………………………………………………..…
5. Email
   ……………………………………………………………………..……………....
6. Home address
   ……………………………………………………………………………………..
7. Date of completing the questionnaire
   ………………………………………………………………………………………
8. Current body weight and height

   Body weight ……….. [kg]   height ……….. [cm]

9. Are you under constant endocrine care?

   (please mark "X" the correct answer) Yes☐ No☐

   If you are, please provide the name of the institution
   ………..…………………………………………………………………………...
   …………………………………………………………………………………………

10. Did you remain under the endocrine care of a center other than CMHI up to 18 years?

    Yes☐ No☐

    If you did, please provide the name of the institution

    …………………………………...…………………………………………………

    ……………………………………………………………………………………...

11. Have you been diagnosed with a recurrence of Cushing's disease? Yes☐ No☐

If you have: What were the symptoms of recurrence of Cushing's disease?
………………………………………………………………………………………..…….

If you have: When did the symptoms of recurrence appear ? (please provide an indicative date of the first symptom)
……………………………………………………………………………………..…

Please provide the date of the recurrence diagnosis.
………………………………………………………………………………………..

How was the recurrence treated? (second surgery?/radiation therapy?/medicines?)
....…...

12. Do you take any medicines? Yes☐ No☐

If you do: what medicine do you take, how many times a day, please provide a dose?
………………………………………………………………………………………
………………………………………………………………………………………
………………………………………………………………………………………

13. Have you been treated with a growth hormone? Yes☐ No☐
If you have: How long? Until when?
…………………………...

14. Have you taken hydrocortisone in the past? Yes☐ No☐
If you have: How long? Until when?
....

15. Have you taken thyroid hormones in the past? Yes☐ No☐
If you have: How long? Until when?
....

16. Have you taken desmopressin in the past? Yes☐ No☐
If you have: How long? Until when?
…………………………...

17. Have you taken testosterone in the past? Yes☐ No☐
If you have: How long? Until when?
………………………...

18. *(concerns woman)* Have you taken estrogens in the past? Yes☐ No☐
If you have: How long? Until when?
………………………...

19. Have you taken dehydroepiandrosterone in the past? Yes☐ No☐

If you have: How long? Until when?
………………………...

20. Do you take metformin or have you taken it in the past? Yes☐ No☐
If you have: How long? Until when?
………………………...

21. Do you have chronic diseases? Yes☐ No☐

If you do, what kind of diseases?

diabetes? Yes☐ No☐

prediabetes? Yes☐ No☐

hypertension? Yes☐ No☐

liver steatosis? Yes☐ No☐

Other diseases? (*please specify)*

…………………………………………………………………………………
…………………………………………………………...…………………………

22. Are you under constant non-endocrine specialist care? Yes☐ No☐

If you are, what and for what reason?
……………………………………………………………………………...………………
……………………………………………………………………………………..

23. Have you undergone neurosurgical operations performed outside the CMHI?
Yes☐ No☐

If you have: what kind of?
……….…………...

when?...

24. Have you had adrenal glands surgery? Yes☐ No☐
If you have: what kind of?
……………………...

when?..

25. Please provide the date of the last ophthalmologic evaluation and its result
Date of examination ……………………………...…………

ophthalmologic assessment: ☐ without deviations in ophthalmological examination

☐ impaired vision acuity

☐ visual field defects

☐ monocular blindness

☐ binocular blindness

26. Do you suffer from osteoporosis? Yes☐ No☐

27. Have you had recently bone mineral density test? Yes☐ No☐

if you have, please provide date of examination and result

…………………………………………………..……………………………………………

28. Have you had any bone fractures? Yes☐ No☐

if you have, when and what kind of fracture it was?
....
....

29. How do you assess your mood?
☐ reduced

☐ normal

☐ elevated

30. Do you have any of the conditions listed below?

Depressive states? Yes☐ No☐

Problems with memory? Yes☐ No☐

Emotional lability? Yes☐ No☐

Problems with concentration? Yes☐ No☐

Mood swings? Yes☐ No☐

Irritability? Yes☐ No☐

31. Do you have a husband / wife / partner / partner? Yes☐ No☐

32. Do you have children? Yes☐ No☐

If you do: how many? ………………………...

33. *(concerns woman)* Do you have difficulties in getting pregnant? Yes☐ No☐

If you do: please write in more detail, e.g. How long does the problem last? Are you taking any medication? Have you plans to try in vitro fertilization?

…………………………………………………………………………………………
…………………………………………………………………………………………

34. Do you have problems with reduced libido? Yes☐ No☐

35. What is your education?

☐ basal

☐ secondary

☐ incomplete higher

☐ higher

Please write in details what school you graduated from

……………………………………………………………………………

36. Please enter your source of income

☐ I work (occupation: …………………………………………………..)

☐ Pension

☐ Im am on a dole

☐ Other………………………………………………………………………………

Do you have any comments to the above questions? Please write below.

…………………………………………………………………………………………
…………………………………………………………………………………………

Thank you for completing the questionnaire!
